# Two Alternating Motor Programs Drive Navigation in *Drosophila* Larva

**DOI:** 10.1371/journal.pone.0023180

**Published:** 2011-08-15

**Authors:** Subhaneil Lahiri, Konlin Shen, Mason Klein, Anji Tang, Elizabeth Kane, Marc Gershow, Paul Garrity, Aravinthan D. T. Samuel

**Affiliations:** 1 Department of Physics, Center for Brain Science, Harvard University, Cambridge, Massachusetts, United States of America; 2 Department of Biology, Brandeis University, Waltham, Massachusetts, United States of America; Alexander Flemming Biomedical Sciences Research Center, Greece

## Abstract

When placed on a temperature gradient, a *Drosophila* larva navigates away from excessive cold or heat by regulating the size, frequency, and direction of reorientation maneuvers between successive periods of forward movement. Forward movement is driven by peristalsis waves that travel from tail to head. During each reorientation maneuver, the larva pauses and sweeps its head from side to side until it picks a new direction for forward movement. Here, we characterized the motor programs that underlie the initiation, execution, and completion of reorientation maneuvers by measuring body segment dynamics of freely moving larvae with fluorescent muscle fibers as they were exposed to temporal changes in temperature. We find that reorientation maneuvers are characterized by highly stereotyped spatiotemporal patterns of segment dynamics. Reorientation maneuvers are initiated with head sweeping movement driven by asymmetric contraction of a portion of anterior body segments. The larva attains a new direction for forward movement after head sweeping movement by using peristalsis waves that gradually push posterior body segments out of alignment with the tail (i.e., the previous direction of forward movement) into alignment with the head. Thus, reorientation maneuvers during thermotaxis are carried out by two alternating motor programs: (1) peristalsis for driving forward movement and (2) asymmetric contraction of anterior body segments for driving head sweeping movement.

## Introduction

Systems neuroscience strives to connect animal behavior to the structure and dynamics of the nervous system. By studying brain and behavior in the *Drosophila* larva, a genetically tractable model organism with a small nervous system and simple body plan, it might be possible to characterize the pathways that encode complex behaviors all the way from sensory input to motor output. To reach this goal, we must develop tools to interrogate all layers of neuronal and muscle dynamics that occur during behavior.

Thermotaxis is a particularly sophisticated behavior exhibited by the *Drosophila* larva [1]–[3]. The navigational strategies of larval thermotaxis were recently analyzed by using a tracking microscope to follow the movements of individual animals responding to defined temperature gradients [4]. In brief, a larva's crawling trajectory can be characterized as a sequence of periods of persistent forward movement (runs) that are interrupted at random by reorientation maneuvers. During a reorientation maneuver, the larva pauses and sweeps its head one or more times until it picks a new direction in which to resume forward movement [5]. Thus, the reorientation maneuvers along an animal's trajectory may be interpreted as a sequence of navigational decisions. In temperature gradients, the larva biases the size, frequency, and direction of these reorientation maneuvers to enhance the likelihood that its overall trajectory trends towards favorable temperatures [4].

Chemotactic and thermotactic navigation in simpler organisms like the bacterium *E. coli* or the nematode *C. elegans* also involves alternating sequences of forward movements and reorientation maneuvers [6]–[8]. However, bacteria and nematodes only bias the frequency of abrupt reorientations in response to changing conditions, exhibiting longer runs when headed in favorable directions and shorter runs when headed in unfavorable directions. By additionally biasing the size and direction of reorientation maneuvers, larvae are able to exhibit more runs in favorable direction, thereby increasing the rate at which they improve their surroundings [4].

The navigational behaviors of the *Drosophila* larva are encoded in neural circuits that activate appropriate motor programs in response to sensory inputs. It is straightforward to estimate sensory input during larval thermotaxis by measuring the position of the larva's body in ambient spatial or temporal gradients. It is less easy to characterize the motor programs that are used during thermotaxis. Because each body segment of the *Drosophila* larva can contract or expand, it is difficult to infer motor dynamics from the outline of the larva's body. Fluorescence microscopy offers a solution: it is possible to visualize the movements of individual segments within transgenic animals in which GFP is fused to the myosin heavy chain [9], [10]. Here, by imaging segment dynamics of freely moving transgenic larvae, we characterize the motor programs that the *Drosophila* larva uses to initiate, execute, and complete reorientation maneuvers during navigation.

## Results

### Visualizing motor dynamics in freely moving larvae

**Figure 1:**
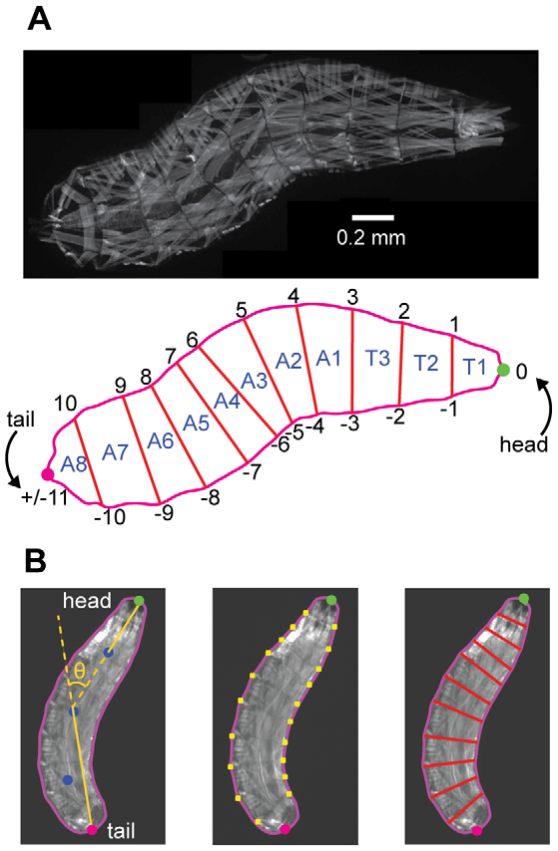
Segmentation of *Drosophila* larva. **A.** (*Upper*) Fluorescence micrograph of *w;Mhc-GFP^c110^/CyO* larva *(Lower)* Coordinate system for delineating the three thoracic (T1…T3) and eight abdominal (A1…A8) segments. The tip of the head and tail are defined as 0 and ±11, respectively. Boundaries between adjacent segments are numbered from +1 to +10 along the left side of the animal and from −1 to −10 along the right side, as shown. **B.** Steps in video analysis. *Left* The boundary, head (green dot), tail (red dot), points along the centerline (blue dots), and operational definition of bend angle used to flag reorientation maneuvers are calculated using machine-vision algorithms described in Materials and Methods. *Center* The twenty points (+1 to +10 and −1 to −10) that define the boundaries between segments are identified by hand (yellow dots). *Right* The lengths of the boundaries between segments or fluorescence intensity within segments can be used as measures of segment contraction.

Transgenic *Drosophila* larva with GFP fused to the myosin heavy-chain (*w;Mhc-GFP^c110^/CyO*) permit the visualization of thoracic and abdominal body segments in the freely moving animal using fluorescence microscopy [9], [10] (Fig. 1A). To facilitate quantification, we developed semi-automated machine-vision software that analyzes the fluorescent images of these crawling larvae to automatically extract the outer boundary, identifies the head and tail, and measures the curvature of the body centerline that is associated with turning decisions (Fig. 1B). Within these image frames, the coordinates of the intersections of the boundaries between each segment with the outer boundary of the animal can be precisely located by hand. Once this coordinate system has been imposed on each video frame, it is straightforward to quantify the contractile dynamics of each of three thoracic body segments (T1…T3) and eight abdominal segments (A1…A8). The mouth segment and the terminal/A9 segment cannot be distinguished in this way due to the lack of sharp boundaries in their musculature.

**Figure 2:**
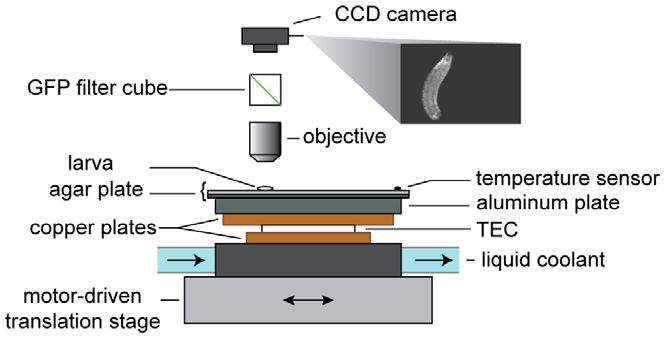
Experimental setup. Spatially uniform, temporal variations in temperature were achieved by controlling the temperature of an aluminum slab with a feedback-controlled thermoelectric controller (TEC). The TEC was coupled to the aluminum slab and liquid-cooled heat sink by copper plates. A motor-driven microscope stage kept each larva within the center of the field of view as it was subjected to defined temperature waveforms as high-resolution video of GFP fluorescence was captured by CCD camera.

**Figure 3:**
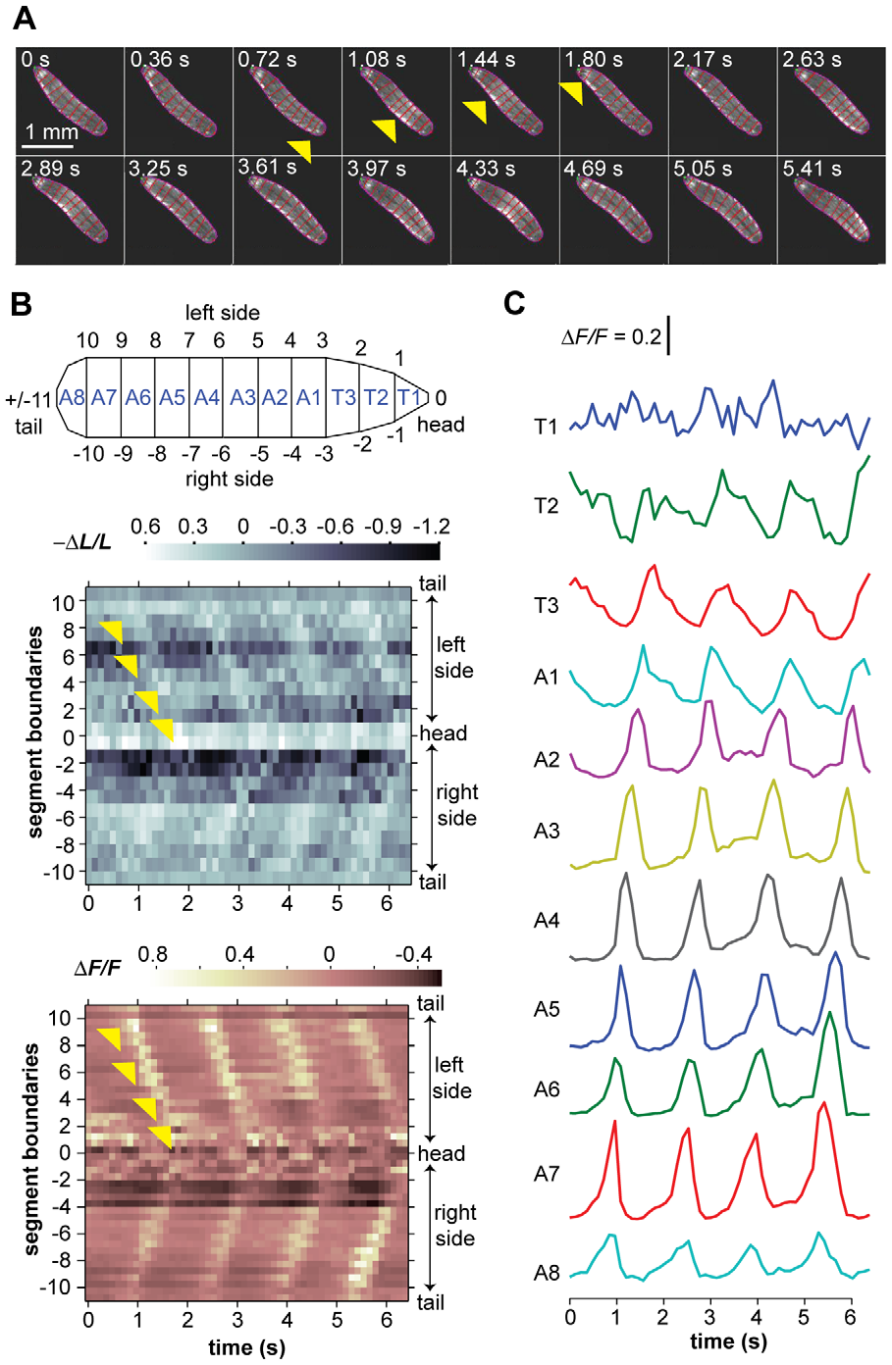
Forward movement. **A.** Video frames showing four peristalsis waves of a larva during forward movement. The larva is kept in the center of the field of view with an automated tracking stage. Outline and boundaries between segments are shown in red, applied using the steps described in Fig. 1B. Yellow arrowheads mark the propagation of the first peristalsis wave from tail to head. **B.** Kymographs showing two measures of body segment contraction. Segment boundaries are used to define the coordinate system along the body as shown in the schematic (also see Fig. **1B**), with integers specifying boundaries between segments from head (0) to tail (±11) along the larva's left side (0 to +11) and right side (0 to −11). (*Upper*) Fractional changes in the lengths of each segment boundary relative to a reference image between peristalsis waves. (*Lower*) Fractional changes in the mean fluorescence intensity within body segments relative to a reference image. In both kymographs, yellow arrowheads mark the propagation of a peak of body segment contraction from tail to head during the first peristalsis wave. **C.** Temporal variations in the mean fluorescence intensity within each body segment taken from the kymograph of **3B.** Vertical scale bar indicates 20% change in fractional fluorescence intensity.

Forward movement of the *Drosophila* larva, as in other *Diptera* species, is driven by waves of peristalsis that travel from tail to head [11]. We began by quantifying body segment dynamics during forward movement, using a tracking microscope that captured high-resolution fluorescence video of freely crawling larvae (Fig. 2; Movie S1). Consistent with previous observations, waves of contraction propagate with nearly constant speed from tail to head during forward movement, starting at the rearmost abdominal segments and ending at the T2 or T1 thoracic segment, each wave producing ∼0.13 body lengths of forward displacement (Fig. 3A) [9], [12].

One measure of segment dynamics during the contraction wave is provided in the lengths of the left and right boundaries of each segment. Another measure is mean fluorescence intensity because muscle contraction increases the local concentration of GFP. Waves of contraction can be graphically represented either with kymographs that show fractional changes in the length of body segment boundaries over time or in the fluorescence intensity of the pixels within each body segment over time (Fig. 3B).

When each body segment is viewed separately, its fluorescence intensity oscillates in a rhythmic manner with successive peristalsis waves. The slight phase shift in the rhythmic oscillation between adjacent segments is due to the propagation of the wave from tail to head. This rhythmic variation in fluorescence intensity is pronounced in the abdominal segments and the T3 thoracic segment, but becomes less noticeable in the T2 segment and especially the T1 segment (Fig. 3C).

**Figure 4:**
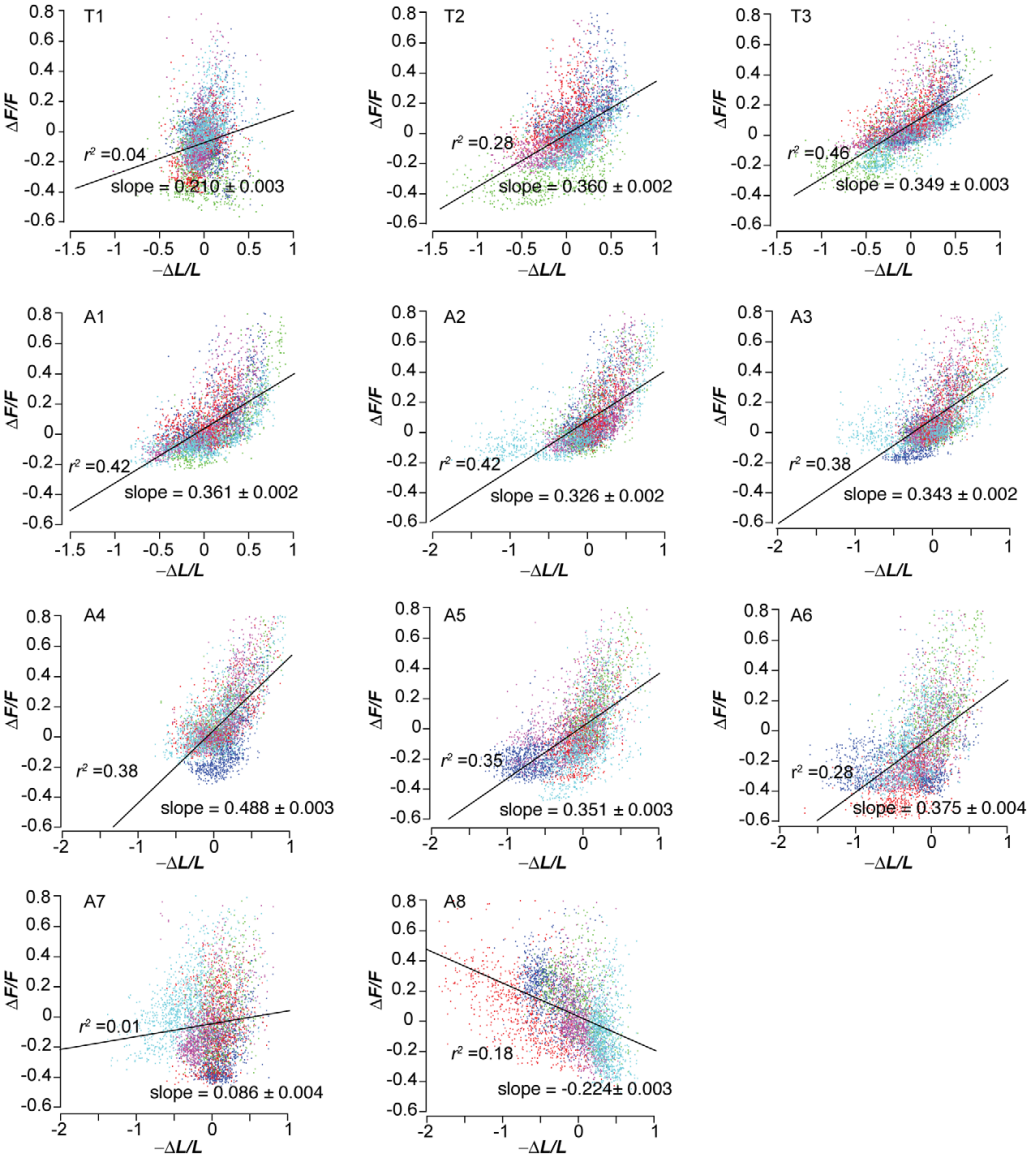
Fractional change in fluorescence intensity vs. fractional change in segment length. Scatter plots of each pair of measurements of each segment's dynamics taken from video of 5 larvae. Data points for each animal are shown in a different color, and each data point represents the analysis of one video frame. Correlation coefficients and the slope ± one standard error for the linear regression (black line) of each scatter plot are shown.

To better understand the two measures of segment contraction, we quantified the correlation between the two sets of measurements across our data sets (Fig. 4). For most segments, increases in fluorescent intensity are strongly correlated with decreases in segment length, as expected. However, at the front, the correlation between fluorescence intensity and segment length becomes smaller in T2 and nearly vanishes in T1. At the rear of the animal, the correlation becomes smaller in A7 and increases in fluorescent intensity become weakly correlated with increases in segment length in A8. The detailed motions of the frontmost and rearmost segments could be more complicated than middle segments. For example, the larva's mouth can exhibit foraging movements, rapid, small amplitude side-to-side movements that seem to be driven by T1. However, the loss of correlation in the two measures of segment contraction at the head and tail could also be partly due to measurement artifact, e.g., owing to the triangular shape of the T1 and A8 segments, measuring the lengths of their left and right sides will be strongly affected by errors in locating their tips.

### Motor dynamics during navigational decision-making

The navigational strategy for larva thermotaxis is based in the regulation of head sweeping movements during the reorientation maneuvers that separate successive periods of forward movement [4]. First and second instar *Drosophila* larvae avoid temperatures below ∼22°C. First, we verified that transgenic *Drosophila* larvae (*w;Mhc-GFP^c110^/CyO*) exhibit cold-avoidance behavior by placing them on spatial temperature gradients. When started at 17°C on linear temperature gradients (0.3°C/cm), both wild-type (*Canton S*) and transgenic larvae crawl towards warmer temperatures at comparable rates (Table 1). The instantaneous crawling speeds of both types of larvae during periods of forward movement are also comparable. Moreover, navigational decisions during thermotaxis are informed by temporal comparisons in temperature. Thus, the frequency of turning decisions and size of head sweeps during turning decisions are greater for cooling ramps than for warming ramps at temperatures around 20°C (Table 1). We verified that both wild-type and transgenic larva exhibit these biases in behavior (Table 1). Taken together, these results suggest that the transgenic larvae do not suffer severe locomotor or navigational impairments. We note that the *white* mutation that is carried by the transgenic larvae has been reported to reduce dopamine and serotonin levels in the larval central nervous system, which may account for differences in the magnitude of the thermosensory response (Table 1) [13]–[15].

**Table 1:** Thermotaxis parameters.

	*Canton S*	*w;Mhc-GFP^c110^/CyO*
*On spatial gradients*		
Thermotaxis speed (mm/s)	0.075±.002	0.055±.002
Mean forward speed during peristalsis (mm/s)	0.32±.01	0.33±.01
*On temporal gradients* Turn frequency (min^−1^)		
Warming	3.2±0.1**	2.6±0.1 **
Cooling	3.8±0.1**	3.8±0.1 **
Size of first head sweep during turns		
Warming	53°±1° *	53°±2° **
Cooling	57°±1° *	60°±2° **

Individual second instar larvae were placed on large Petri plates (20 cm×20 cm) either with superposed spatial temperature gradient or spatially uniform temporal gradient and tracked using similar methods as described elsewhere [4], [24]. At least 20 animals were used for each measurement. Each measurement represents the mean +/− one standard error. On spatial gradients (0.3°C/cm), individual larvae were initially placed near 17°C, and their trajectories towards higher temperatures were analyzed. Thermotaxis speed indicates the mean rate of ascent up the temperature gradient towards warmer temperatures. Mean forward speed during periods of persistent forward movement was also measured. On temporal gradients, larvae were subjected to sinusoidal temperature waveforms, ∼20°C offset, ∼1°C amplitude, 6 min period. The frequency of turning decisions and the mean size of the first head sweep in each turning decision was quantified during both warming and cooling phases of the temperature stimulus. * and ** indicates case in which each statistic differs between warming and cooling at P<0.05 and P<0.005, respectively.

To visualize muscle dynamics during navigational decisions, we subjected individual transgenic larvae to sinusoidal variations between 14°C and 16°C with 2 min period while tracking their movements using the experimental setup shown in Fig. 2. Here, we analyzed 46 abrupt turns exhibited by 10 transgenic larvae that were individually tracked in the new setup, 26 that occurred during the cooling phase and 20 that occurred during the warming phase of the sinusoidal stimulus. We designated turns as *large* (9 of which occurred during warming and 13 during cooling) or *small* (11 of which occurred during warming and 13 during cooling) when the body bend angles were greater or smaller than 90°, respectively.

**Figure 5:**
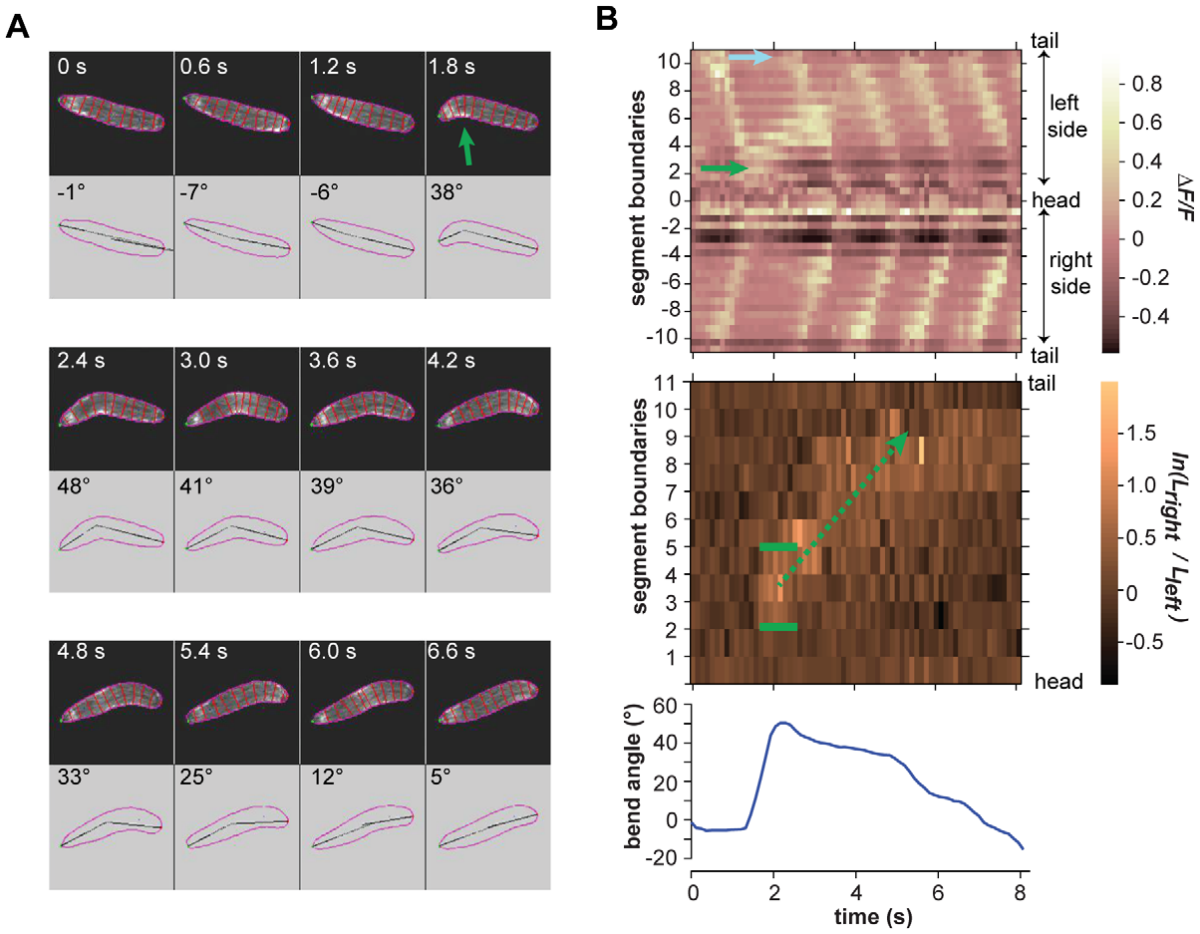
Turning decisions with small head sweeps. **A.** Video frames of an example small head sweep during warming. Upper panel in each video frame shows fluorescence image. Outline and boundaries between segments are shown in red. Lower panel in each video frame shows the perimeter, centerline, and body bend angle as analyzed by machine vision software. Green arrow shows the asymmetric bend that is flagged as a head sweep. **B.** *(Upper panel)* Kymograph of fluorescence intensity within body segments for the video segment shown in **A.** Green arrow shows the increase in fluorescence intensity on the left side of the animal, signifying the leftward head sweep. Blue arrow shows the increase in fluorescence intensity in the A8 segment, signifying the resumption of peristalsis at the tail. *(Middle panel)* Kymograph of the logarithm of the ratio between the lengths of segment boundaries on the left side and right side of the animal. Green brackets indicate the margins of the region of asymmetric contraction, positive values signifying the leftward bend. Green arrow shows the retrograde progression of the region of asymmetric contraction during the four subsequent peristalsis waves. *(Lower panel)* Time course of body bend angle, operationally defined to flag reorientation maneuvers as described in Materials and Methods, shows the initial rise that signifies the leftward head sweep and gradual decline as subsequent peristalsis waves straighten the body.

**Figure 6:**
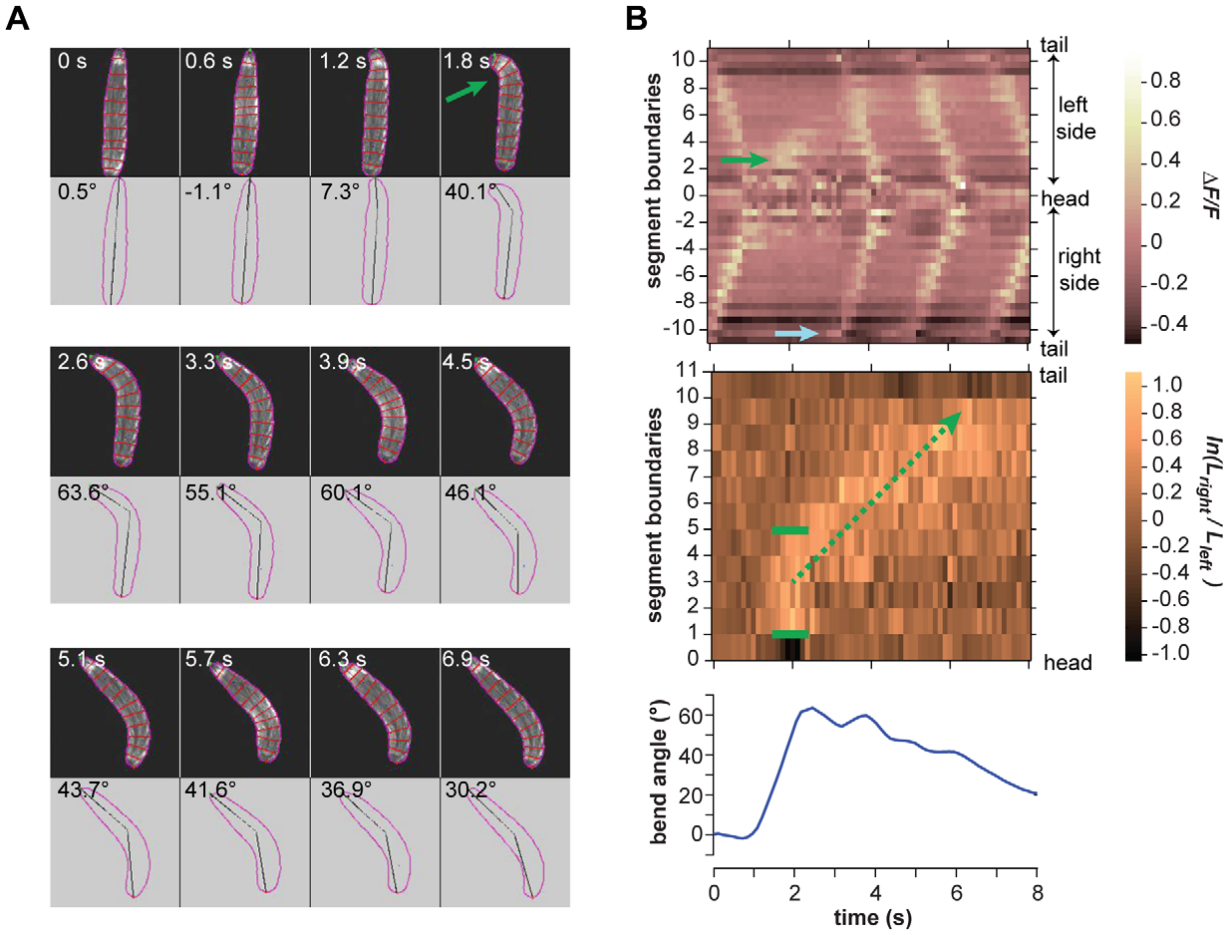
Turning decisions with small head sweeps. **A.** Video frames of an example small head sweep during cooling. Upper panel in each video frame shows fluorescence image. Outline and boundaries between segments are shown in red. Lower panel in each video frame shows the perimeter, centerline, and body bend angle as analyzed by machine vision software. Green arrow shows the asymmetric bend that is flagged as a head sweep. **B.** *(Upper panel)* Kymograph of fluorescence intensity within body segments for the video segment shown in **A.** Green arrow shows the increase in fluorescence intensity on the left side of the animal, signifying the leftward head sweep. Blue arrow shows the increase in fluorescence intensity in the A8 segment, signifying the resumption of peristalsis at the tail. *(Middle panel)* Kymograph of the logarithm of the ratio between the lengths of segment boundaries on the left side and right side of the animal. Green brackets indicate the margins of the region of asymmetric contraction, positive values signifying the leftward bend. Green arrow shows the retrograde progression of the region of asymmetric contraction during the four subsequent peristalsis waves. *(Lower panel)* Time course of body bend angle, operationally defined to flag reorientation maneuvers as described in Materials and Methods, shows the initial rise that signifies the leftward head sweep and gradual decline as subsequent peristalsis waves straighten the body.

**Figure 7:**
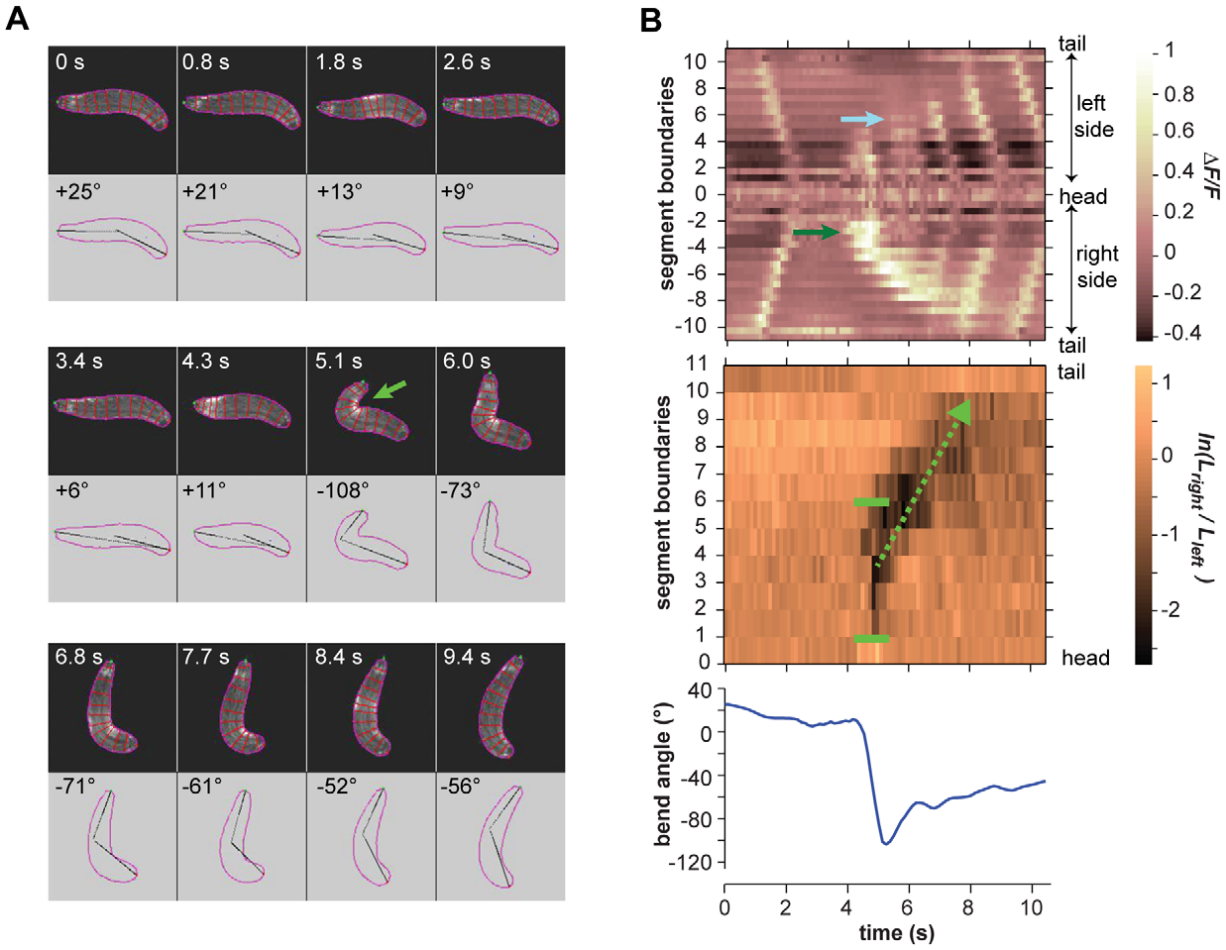
Turning decisions with large head sweeps. **A.** Video frames of an example large rightward head sweep during cooling. Upper panel in each video frame shows fluorescence image. Outline and boundaries between segments are shown in red. Lower panel in each video frame shows the perimeter, centerline, and body bend angle as analyzed by machine vision software. Green arrow shows the asymmetric bend that is flagged as a head sweep. **B.** *(Upper panel)* Kymograph of fluorescence intensity within body segments for the video segment shown in **A.** Green arrow shows the greater increase in fluorescence intensity on the right side of the animal, signifying the rightward head sweep. Blue arrow shows the increase in fluorescence intensity in the A3 segment, signifying the resumption of peristalsis near the bend. *(Middle panel)* Kymograph of the logarithm of the ratio between the lengths of segment boundaries on the left side and right side of the animal. Green brackets indicate the margins of the region of asymmetric contraction, negative values signifying the rightward bend. Green arrow shows the retrograde progression of the region of asymmetric contraction during subsequent peristalsis waves. *(Lower panel)* Time course of body bend angle, operationally defined to flag reorientation maneuvers as described in Materials and Methods, shows the initial drop that signifies the rightward head sweep and gradual rise as subsequent peristalsis waves straighten the body.

**Figure 8:**
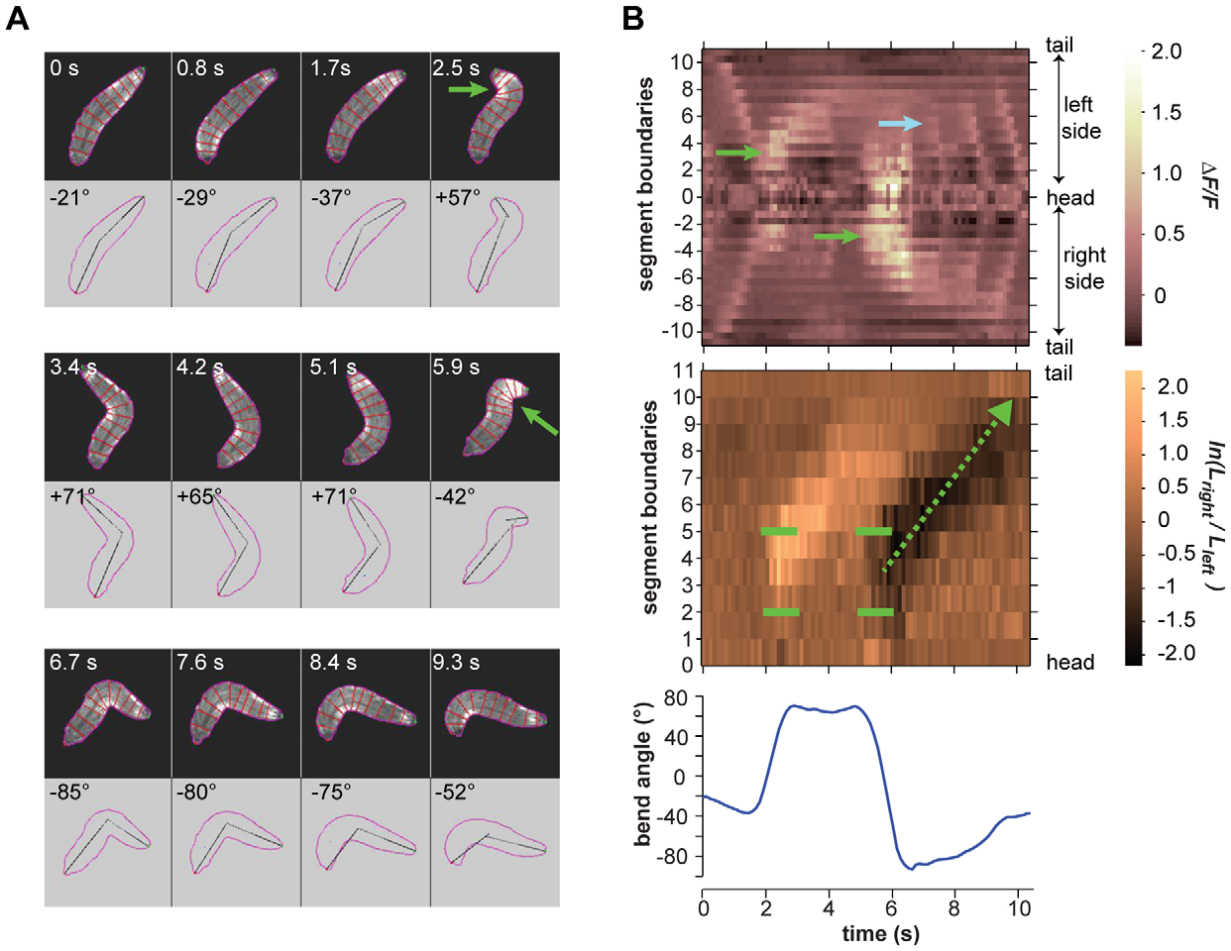
Turning decisions with multiple head sweeps. **A.** Video frames of a turning decision during cooling in which two head sweeps were executed in rapid succession. Upper panel in each video frame shows fluorescence image. Outline and boundaries between segments are shown in red. Lower panel in each video frame shows the perimeter, centerline, and body bend angle as analyzed by machine vision software. Green arrows show the asymmetric bends that are flagged as head sweeps. **B.** *(Upper panel)* Kymograph of fluorescence intensity within body segments for the video segment shown in **A.** Green arrows show the greater increase in fluorescence intensity, first on the left side then the right side of the animal, signifying the leftward and rightward head sweeps. Blue arrow shows the increase in fluorescence intensity in the A3 segment, signifying the resumption of peristalsis near the bend.*(Middle panel)* Kymograph of the logarithm of the ratio between the lengths of segment boundaries on the left side and right side of the animal. Green brackets indicate the margins of the region of asymmetric contraction, positive values signifying the leftward bend and negative values signifying the rightward bend. One peristalsis wave occurs after the leftward head sweep. Green arrow shows the retrograde progression of the region of asymmetric contraction that straighten the body during three peristalsis waves after the rightward head sweep. *(Lower panel)* Time course of body bend angle, operationally defined to flag reorientation maneuvers as described in Materials and Methods, shows the initial rise that signifies the leftward head sweep and drop that signifies the subsequent rightward head sweep.

Video segments and analysis of four example turning decisions are shown in Figs. 5, 6, 7, 8. A reorientation maneuver involving one small leftward head sweep during warming is shown in Fig. 5 (Movie S2). A turning decision involving one small leftward head sweep during cooling is shown in Fig. 6 (Movie S3). A reorientation maneuver involving one large rightward head sweep during cooling is shown in Fig. 7 (Movie S4). A reorientation maneuver involving two large head sweeps in rapid succession during cooling, one leftward and one rightward, is shown in Fig. 8 (Movie S5). Below we discuss how comparison of the time-varying posture of each larva with corresponding kymographs of segment contraction were used to reveal the motor sequences that correspond to the initiation, execution, and completion of reorientation maneuvers.

In every case, we found that the onset of head sweeping during reorientation maneuvers (identified as events in which the body centerline bends by more than 40°) occurs after the end of the previous peristalsis cycle (Fig. 5 and Movie S2; Fig. 6 and Movie S3; Fig. 7 and Movie S4; Fig. 8 and Movie S5). This observation suggests a distinct transition from the peristaltic motor program for forward movement to the asymmetric contraction motor program for initiating head sweeping for each reorientation maneuver.

Next, we looked at how body segments are used to actuate head sweeps. To do this, we quantified asymmetric contraction by measuring the logarithm of the length ratio between the left and right boundaries of the opposing quadrants within each body segment: ln(*L_right_/L_left_*). This logarithm is roughly zero during the peristalsis that accompanies forward movement because the left and right sides of each segment contract and expand simultaneously. During head sweeps, the logarithm of the length ratio of the body segments within the pivot will increase to positive values or decrease to negative values for leftward and rightward sweeps, respectively.

**Figure 9:**
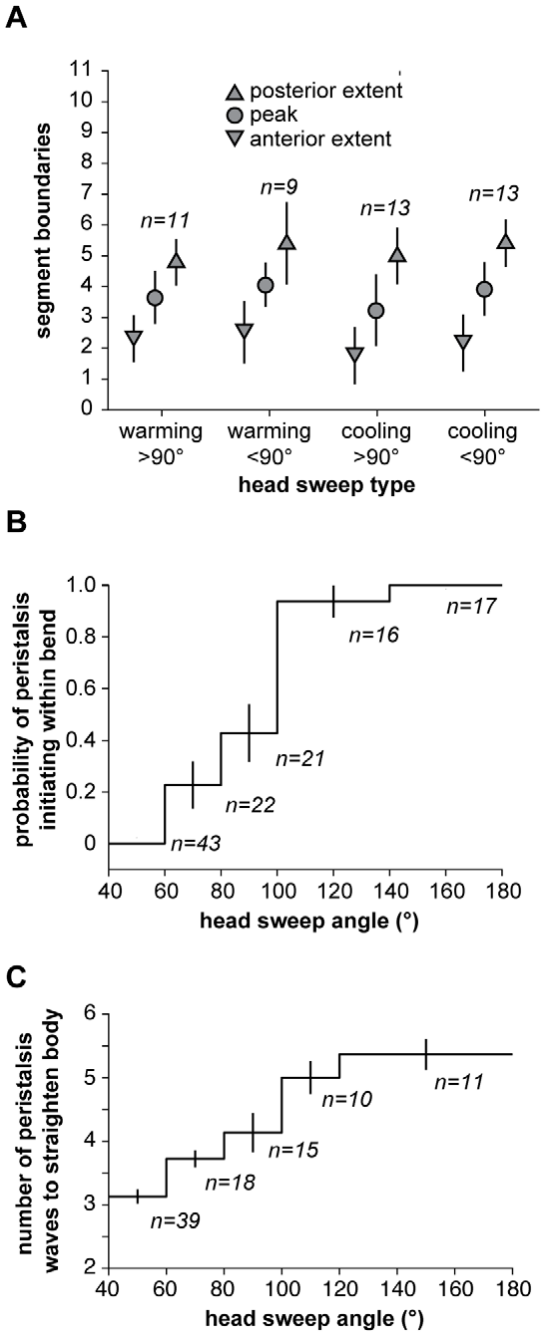
Consistency in segment dynamics during turning decisions. **A.** Region of asymmetric contraction for large (>90°) and small (<90°) head sweeps during warming and cooling. For different classes of head sweeps, the anterior extent (inverted triangle), posterior extent (triangle) and peak position of asymmetric contraction (circle) were flagged in kymographs as shown in Fig. 5B, 6B, 7B, and 8B. Data points represent the mean +/− s.d. of each set of measurements. **B.** The probability of initiating peristalsis waves within the bent portion of the larva increases with the size of head sweeps. The number of measurements for each data point is shown in italics. Error bars represent one standard error. **C.** Larger reorientations require more peristalsis waves to straighten the larva's body. The number of measurements for each data point is shown in italics. Error bars represent one standard error.

We found remarkable consistency in the usage of segments during head sweeps. Neither the anterior and posterior extents of asymmetric contraction, nor the center of the pivot, depended on the size or direction of head sweeps, whether they were initiated during the cooling or warming phases of thermosensory input, or whether one or more head sweeps were involved in each turning decision (Fig. 5 and Movie S2; Fig. 6 and Movie S3; Fig. 7 and Movie S4; Fig. 8 and Movie S5; Fig. 9A). All head sweeps are centered on the A1 segment, which undergoes the peak asymmetric contraction between the left and right sides. The T3, A2 and A3 segment, although exhibiting smaller amounts of asymmetric contraction than A1, also appear to contribute to the pivot (Fig. 9A). Taken together, these observations suggest parsimony in the higher-order commands that evoke head sweeps in turning decisions. The brain only needs to tell one group of segments (T3 through A3) how much to bend.

We found that larvae complete reorientation maneuvers by using peristalsis, gradually straightening their bodies along the new direction for forward movement. When peristalsis resumes after small head sweeps (body bend angles <90°), the first wave of contraction tends to be initiated at the tail (Fig. 5 and Movie S2; Fig. 6 and Movie S3; Fig. 9B), just as in normal forward movement (Fig. 2). With each subsequent wave of contraction, all of which continue to be initiated at the tail, ∼2 additional body segments make their way around the bend. Thus, the region of asymmetric contraction gradually propagates posteriorly, starting from the initial bend at T3-A3 and moving toward the tail until, after 3–4 peristalsis wave, the entire larva has made its way around the bend (Fig. 5B; Fig. 6B). At this point, the straightened larva continues forward movement in its new direction.

When peristalsis resumes after large head sweeps, the first wave of contraction tended to be initiated near the region of asymmetric contraction (Fig. 7 and Movie S4). Thus, the peristalsis of the portion of the larva that is anterior to the pivot appears to pull posterior segments into the region of asymmetric contraction. Each subsequent wave of contraction works the same way, being initiated within the bent portion of the larva, gradually pulling body segments around the original pivot point. After 4–6 peristalsis cycles, the entire larva makes its way around the original pivot point with a new direction for forward movement with straightened body (Fig. 7 and Movie S4).

As shown in Fig. 8 and Movie S5, a reorientation maneuver can be said to involve multiple head sweeps when they are initiated in rapid succession without enough intervening peristalsis waves to straighten the larva body. Thus, in the maneuver shown in Fig. 8 and Movie S5, in which only one peristalsis wave is initiated between the head sweeps, the direction chosen by the first head sweep was “rejected” whereas the direction chosen by the second head sweep was “accepted” and became the direction of the subsequent run. Earlier it was shown that the probability of rejecting a head sweep during larval cold-avoidance behavior was raised (/lowered) if the larva experienced cooling (/warming) during the head sweep [4].

By examining the resumption of peristalsis after head sweeps of different size, we found that the first wave of contraction was always initiated either at the tail (more probable for smaller head sweeps) or within the region of asymmetric contraction (more probable for larger head sweeps) (Fig. 9B). In addition, more peristalsis cycles are required to straighten the larva after larger head sweeps (Fig. 9C).

## Discussion

Animals execute complex behaviors by breaking tasks into discrete steps that can be accomplished through the deployment of motor programs. Understanding orientation behavior, how an animal senses and moves with respect to stimulus gradients in its environment, has long been a classic problem in neuroethology [16]. Classic studies in the phototactic behavior of maggot larvae, for example, identified and explored how movement patterns like head sweeping allow these small animals to change their direction of movement with respect to light sources [5]. However, comprehensive understanding of the neural basis of orientation behavior necessitates using genetically and physiologically tractable model organisms like *Drosophila*.

In an earlier study, we showed that decision-making during larva thermotaxis occurs during the reorientation maneuvers that separate successive periods of forward movement [4]. Here, we used the direct visualization of segment dynamics with high-resolution fluorescence microscopy to characterize the motor programs that drive thermotaxis in transgenic *Drosophila* larvae with fluorescently labeled muscle fibers. We show that the initiation, execution, and completion of these reorientation maneuvers are carried out by alternating deployment of two types of motor programs: one for asymmetric contraction that drives anterior bending (head sweeping) and one for peristalsis that drives forward movement. The high degree of stereotypy in each motor program suggests that navigational strategy can be executed with a relatively small set of commands that emanate from the larval brain. First, the brain uses thermosensory input to trigger the transition from peristaltic forward movement to head sweeping movement that signifies the onset of each reorientation maneuver. During the initial bending movements of a head sweep, the brain specifies the amplitude of each head sweep to be carried out by one group of body segments. Finally, to complete the reorientation maneuver, the larva returns to the motor program for peristalsis to straighten the body along the new direction for forward movement.

We have also seen that, to complete a turn, peristalsis can resume from different positions depending on the size of the head sweep. It is unclear why this happens, but our observations do demonstrate that peristalsis can be initiated at different points along the body. One reason might be to avoid disrupting the angle of reorientation defined by the angular size of large head sweeps: if peristalsis were initiated at the tail after a large head sweep, the position of the pivot might be pushed forward when the peristalsis reaches the pivot, perhaps increasing the angle of the reorientation beyond the size of the original head sweep. The ability to initiate peristalsis in different segments depending on the degree of body bend might point to a role for sensory feedback in the motor circuit. Proprioceptive feedback has recently been shown to play an essential role in generating peristalsis in the *Drosophila* larva [10], [17], [18].

*Drosophila* larvae also exhibit phototaxis and chemotaxis, but the navigational strategies have yet to be defined in the same detail as larval thermotaxis [19]–[22]. Comparative analysis of navigational strategies and motor programs during different modes of navigation would illuminate whether shared sensorimotor pathways are used during different navigational modes. Here, we focused on the two motor programs that build thermotaxis, but other motor programs appear to be used during other types of behavioral response, such as reverse crawling, hunching, and rolling movements that are used during nociceptive and rapid avoidance responses [21]. Elucidating the complete set of motor programs that can be carried out by the *Drosophila* larva will yield the building-blocks of its total behavioral repertoire. Elucidating the pathways by which these motor programs are triggered by environmental stimuli will yield a complete understanding of brain and behavior in this small animal.

We note that the progression of work on thermotactic navigation in the *Drosophila* larva parallels earlier studies of bacterial chemotactic navigation. The biased-random walk strategy of bacterial chemotaxis was originally established by using a tracking microscope to follow the movements of individual bacteria as they navigated chemical gradients: *E. coli* swims by alternating periods of forward movement (runs) with erratic reorientation movements (tumbles), and the bacterium postpones tumbles when it senses increasing amounts of chemoattractant in its surroundings [6]. A tracking microscope was also used to establish that the larva navigates by alternating periods of forward movement with turning decisions [4]. A recent study of bacterial chemotaxis, which parallels this study, used advances in fluorescent labeling and video microscopy to directly visualize how the detailed movements of individual bacterial flagella contribute to the initiation, execution, and conclusion of tumbles [23]. Both bacteria and *Drosophila* larva use essentially two motor programs to navigate. By regulating the transition from forward movement to reorientation movement in response to sensory conditions, both organisms lengthen runs that happen to be pointed in favorable directions. The head sweeping that characterizes navigational decisions during larval thermotaxis allows the animal to explore an additional axis during reorientation, enabling it to point more runs towards favorable directions. The *Drosophila* larva does not improve its navigation beyond bacterial navigation by using a larger repertoire of motor programs, but by using both motor programs to more efficiently assess preferred directions in its environment.

## Materials and Methods

### Fly strains

To quantify the parameters of thermotactic strategy as shown in Table 1 we followed methods described in our earlier study [4]. Our wild-type strain was *Canton S*. To quantify muscle dynamics, we used *w;Mhc-GFP^c110^/CyO* second instar larvae. Larvae were raised at 25°C on grape juice plates with yeast paste. Individual larvae were removed from the plates, rinsed in distilled water, and transferred to the agar surface of the tracking and imaging setup shown in Fig. 2.

### Image acquisition and tracking

Individual second instar larvae were placed on flat agar surfaces. The agar (1% agarose in distilled water) had been poured onto thin black anodized aluminum plates (8.3×8.3×0.16 cm), forming a layer approximately 2 mm thick. The square agar plates were placed under a microscope (Eclipse LV100, Nikon), and blue light (450–490 nm) was shone onto a single larva, with the resulting GFP signal (500–550 nm) from larval muscles imaged onto a CCD camera (CoolSNAP EZ, Photometrics), with images acquired at 8 Hz. Unrestrained larvae were free to crawl and exhibit behavior on the agar surface, and were kept in the field of view of the camera via feedback to a motorized x-y stage (MAC6000, Ludl Electronic Products). Image acquisition, motorized stage feedback, and temperature control (see below) were all integrated into customized software written in LabVIEW (National Instruments). For each acquired image, a binary-thresholded version was generated, with the larval center of mass position relative to the image center used to determine the necessary direction and magnitude of the stage movement. Raw larval images, stage positions, and frame times were all recorded.

### Temperature control

Approximate larval temperature during experiments was measured using a K-type thermocouple embedded just under the agar surface. The temperature was controlled with a custom-built feedback circuit consisting of a PID controller and H-bridge amplifier (FTC200 and FTX700, Ferrotec) driving a TEC device (CP1-12710, Thermal Enterprises). The TEC pumped heat (in either direction) between a copper block with the stage and agar surface atop, and a circulating liquid coolant reservoir. This setup allowed rapid heating and cooling of the larva being tracked, and its response to temperature changes could be readily discerned. Temperature was sine-wave modulated, with 1°C amplitude and 2 min period with 15°C offset. Note that the temporal gradients that we used on the high-resolution imaging setup differed from the temporal gradients described in Table 1, where we used higher absolute temperatures and shallower gradients to compare the cold-avoidance response of the transgenic strain to our wild-type strain.

### Image analysis

Image analysis operations illustrated in Fig. 1B were performed with custom software written in MATLAB. For each video frame, the boundary of the larva and positions of head and tail were located as follows. First, the image was thresholded to produce a binary image. The binary image was smoothed using erosion and dilation, image processing filters available in MATLAB. The largest boundary in the binary image corresponding to the perimeter of the larva body was determined, and further smoothed with a Gaussian filter with width corresponding to 0.5% of boundary length. The curvature of the boundary was computed with a sliding window (20% of the total boundary length), and the head and tail were identified as the two local maxima. The head and tail were distinguished from each other by proximity to their locations in the previous frame. In the first frame of each video, the distinction was made with user input.

Next, an operational definition of body bend angle was used to detect the onset of head sweeps. First, we identified the body centerline as the midpoints of the lines joining corresponding points on the left and right of the larva body. Three points were marked along the body centerline at ¼, ½, and ¾ of total length (Figure 1B). We quantified body bend as the angle between the line segment joining the tail to the midpoint and the line segment joining the head to the ¾-point (marked as θ in Figure 1B). Leftward and rightward head sweeps are indicate by positive and negative angles, respectively. We flagged each head sweep when the magnitude of this angle exceeded 40°. Note that this definition of body bend angle will only be accurate when the bend lies between the ¾-point and the midpoint, but suffices for flagging the beginning and end of reorientation maneuvers.

Finally, in each video frame the user clicked on the 20 points where segment boundaries intersect the boundary of the larva. This operation split the larva image into three thoracic (T1…T3) and eight abdominal (A1…A8) segments. The left and right boundaries of each segment could be directly quantified as a metric of segment contraction. Alternatively, each segment was split into four quadrants (anterior left, anterior right, posterior left, posterior right) so that fluorescence intensity could be computed from the mean pixel value in each quadrant. The points between segment boundaries were used to define the coordinate system for analyzing body segment dynamics as shown in the schematic of the larva body in Fig. 3B.

### Statistical procedures

In Figure 4, the slope of the best fit line and correlation coefficient, *r* ^2^, were computed with Ordinary Least Squares simple linear regression using MATLAB.

In Figure 9A, for each head sweep, the anterior and posterior extent of asymmetric contraction, as well as the position of peak asymmetry were recorded. The mean and standard deviations were computed using MATLAB. In Figure 9B, for each head sweep, the peak body bend angle and whether or not peristalsis reinitiated from the bent region was recorded. Within each bin for the angle, the total number of head sweeps, *n_i_*, and the number of times peristalsis reinitiated from the bent region, *m_i_* , were used to compute probability and standard error.

In Figure 9C, for each head sweep, the peak body bend angle and the number of peristalsis pulses needed was recorded. Within each bin for the angle, the mean and standard deviations were computed using MATLAB.

For Table 1, thermotaxis speed on spatial gradients was calculated by taking the mean position of the population as a function of time and performing a linear fit of the resulting data. The population position stabilized as larvae reached their preferred temperature; data for times after this occurred were not used. For mean forward speed during peristalsis, we averaged the speed of larvae at each acquired image throughout the recorded movie, excepting larvae in the midst ofturning decisions.

For temporal gradient thermotaxis, we defined warming (/cooling) to occur when the rate of change of the agar surface temperature was greater (/less) than 0.001°C/sec. We counted the total number of reorientations occurring during heating and cooling, then divided by the total time that warming and cooling occurred during an experiment, which yielded the turn frequency. We also examined the head sweeps that occurred during reorientation events, extracting the angle of the first head sweep, and then averaging all such events for both warming and cooling reorientations.

## Supporting Information

Movie S1 Right panel shows video of freely crawling larva corresponding to the video frames shown in Fig. 3A. Left panel shows kymograph of fractional changes in fluorescence intensity during the video as shown in lower panel of Fig. 3B.
(MOV)

Movie S2 Right panel shows video of larva exhibiting a small leftward head sweep corresponding to the video frames shown in Fig. 5A. Upper left panel shows kymograph of fractional changes in fluorescence intensity as shown in upper panel of Fig. 5B. Lower left panel shows kymograph of the logarithm of the ratio between right and left boundary lengths of each segment as shown in the middle panel of Fig. 5B.
(MOV)

Movie S3 Right panel shows video of larva exhibiting a small leftward head sweep corresponding to the video frames shown in Fig. 6A. Upper left panel shows kymograph of fractional changes in fluorescence intensity as shown in upper panel of Fig. 6B. Lower left panel shows kymograph of the logarithm of the ratio between right and left boundary lengths of each segment as shown in the middle panel of Fig. 6B.
(MOV)

Movie S4 Right panel shows video of larva exhibiting a large rightward head sweep corresponding to the video frames shown in Fig. 7A. Upper left panel shows kymograph of fractional changes in fluorescence intensity as shown in upper panel of Fig. 7B. Lower left panel shows kymograph of the ratio between right and left boundary lengths of each segment as shown in the middle panel of Fig. 7B.
(MOV)

Movie S5 Right panel shows video of larva exhibiting a leftward then rightward head sweep corresponding to the video frames shown in Fig. 8A. Upper left panels shows kymograph of fractional changes in fluorescence intensity as shown in upper panel of Fig. 8B. Lower left panels shows kymograph of the ratio between right and left boundary lengths of each segment as shown in the middle panel of Fig. 8B.
(MOV)
